# Supplementary material for: Intratumoral delivery of FLT3L with CXCR3/CCR5 ligands promotes XCR1+ cDC1 infiltration and activates anti-tumor immunity
Source: Nat Commun. 2025 Dec 30;17:1258. doi: 10.1038/s41467-025-68018-3 (PMC12865026; doi:10.1038/s41467-025-68018-3)
Supplement: Supplementary file 1 — Supplementary Information [file 41467_2025_68018_MOESM1_ESM.pdf]

Supplementary Information

Intratumoral delivery of FLT3L with CXCR3/CCR5 ligands promotes XCR1^{+} cDC1 infiltration and activates anti-tumor immunity.

Supplementary information includes:

Supplementary Figures 1-10

Supplementary Table 1 – Resource table.

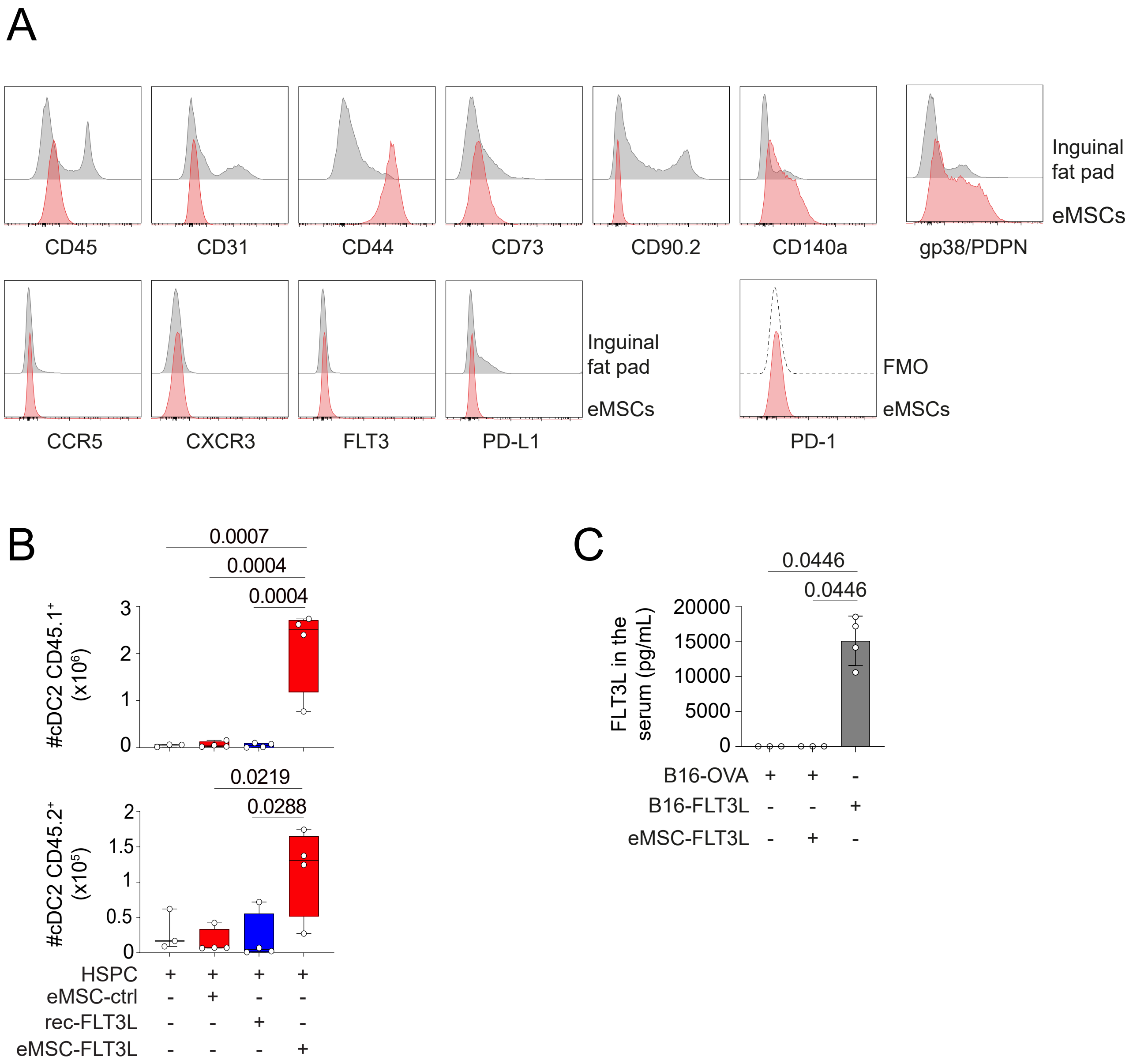

Supplementary Figure 1: Phenotypic characterization and biological activity of eMSCs.

(A) Histograms showing flow cytometry phenotypic analysis of eMSCs or inguinal fat pad cells using various classical and conventional markers for stromal and stem cells. Representative of two independent experiments.

(B) Quantification at day 12 of CD45.1 and CD45.2 cDC2s in the synthetic niches containing HSPC only (n=3 plugs) or with eMSC-FLT3L, eMSC-Ctrl or recombinant FLT3L (rec-FLT3L) (n=4 plugs), one experiment, one-way ANOVA-test with Tukey's multiple comparisons. Box plots show median, 25th–75th percentiles, minimum–maximum whiskers, with all data points displayed.

(C) Circulating FLT3L levels measured by ELISA in the serum of mice bearing B16-OVA (n=3), B16-OVA+eMSC-FLT3L (n=3) or B16-FLT3L (n=4), 4 days after eMSCs injection, one experiment, Kruskal-Wallis test with Dunn's multiple comparisons. Data are presented as mean values ± SD.

(B-C) Source data are provided as a Source Data file.

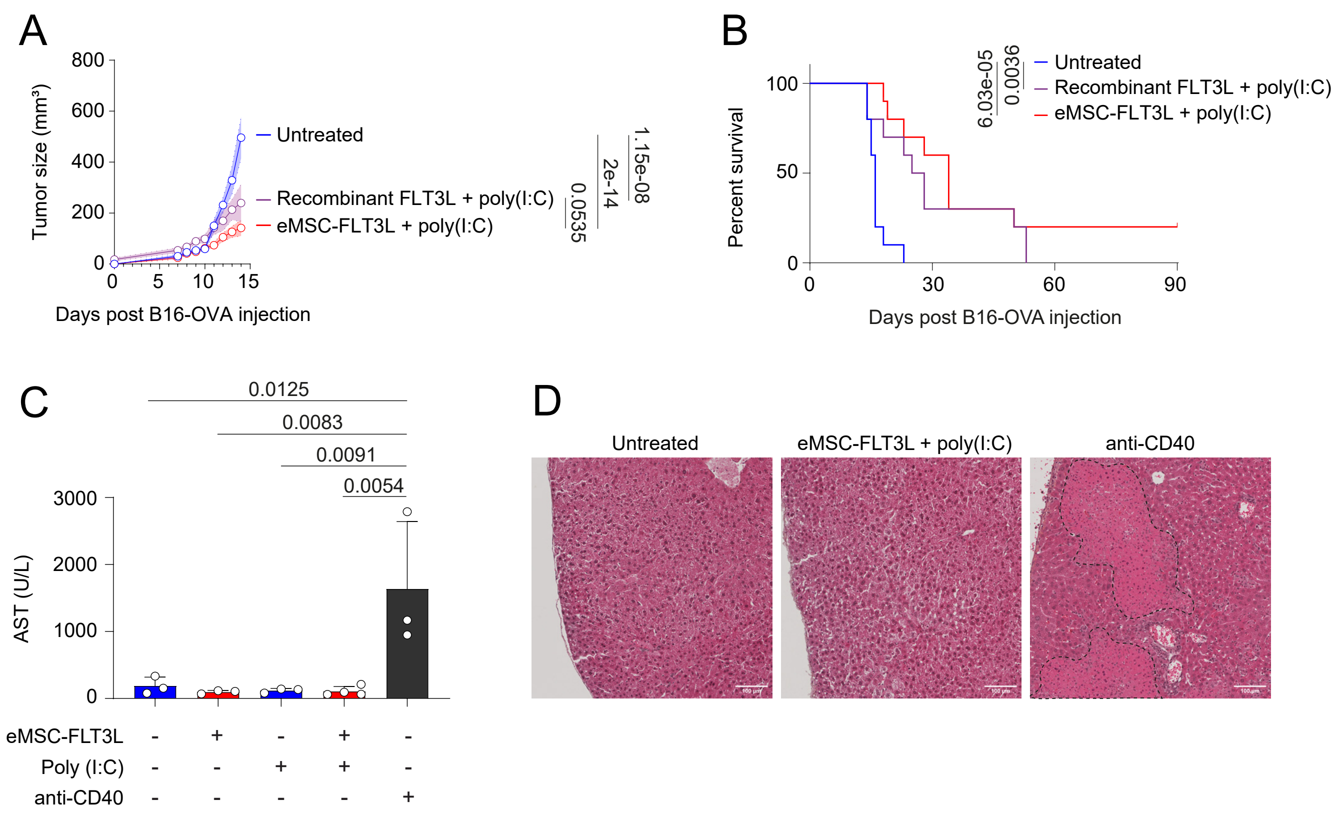

Supplementary Figure 2: eMSC-FLT3L + poly(I:C) immunotherapy is more effective than recombinant FLT3L + poly(I:C) treatment, without inducing toxicity.

(A) Tumor growth curves until day 14, n=10 mice per group, two independent experiments, two-way ANOVA-test with Tukey's multiple comparisons test. A line represents the mean and SEM is shown with the colored area.

(B) Survival curves, n=10 mice per group, two independent experiments, log-rank (Mantel-Cox) test.

(**C**) Level of AST in serum collected 48h after poly(I:C) or anti-CD40 treatment, n=4 (eMSC-FLT3L+poly(I:C)), n=3 mice (other ones), one experiment, one-way ANOVA-test with Tukey's multiple comparisons. Data are presented as mean values ± SD.

(**D**) H&E staining of fixed liver tissue from untreated mice (n=3) or mice treated with eMSC-FLT3L+poly(I:C) therapy (n=4) or anti-CD40 (n=4), one experiment. Necrotic lesions are shown with the dashed black lines.

(A-C) Source data are provided as a Source Data file.

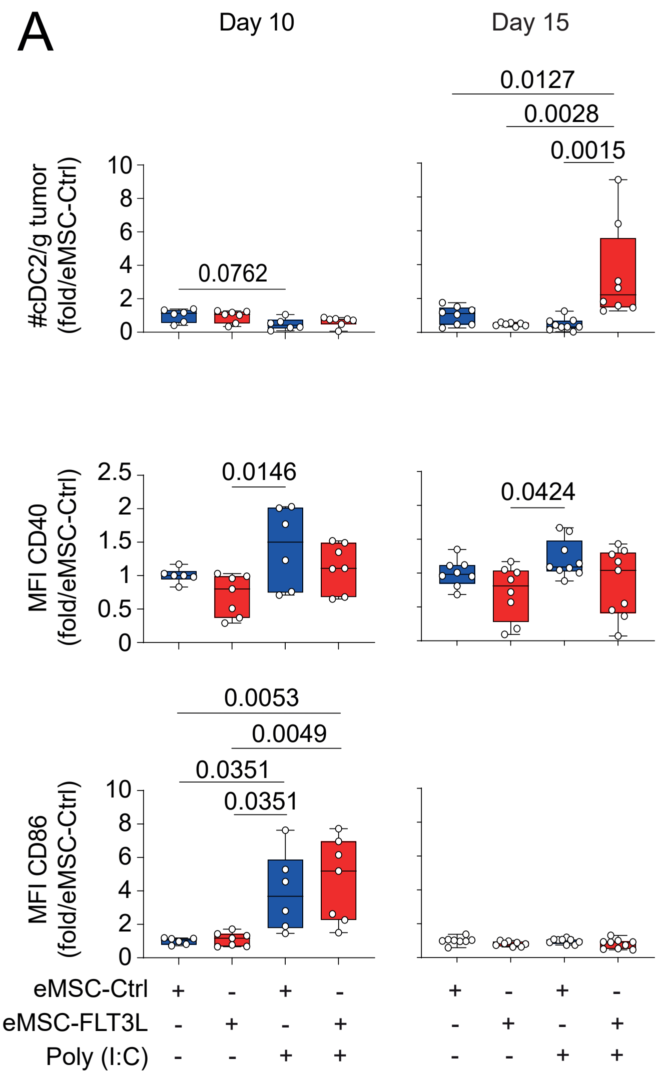

<u>Supplementary Figure 3:</u> Intratumoral delivery of eMSC-FLT3L + poly(I:C) stimulates cDC2 infiltration within tumor.

(A) Quantification of the absolute number of intratumoral cDC2/g tumor and the mean fluorescence intensity (MFI) of CD40 and CD86. Results are shown as fold change to control (eMSC-Ctrl). Day 10: n=6 (eMSC-Ctrl, eMSC-Ctrl+poly(I:C)), n=7 (eMSC-FLT3L, eMSC-FLT3L+poly(I:C)) mice per group, two independent experiments, one-way ANOVA-test with Tukey's multiple comparisons. Day 15: n=8 (eMSC-Ctrl, eMSC-FLT3L+poly(I:C)), n=7 (eMSC-FLT3L), n=9 (eMSC-Ctrl+poly(I:C) mice per group for the number of cDC2s/g tumor; n=8 (eMSC-Ctrl, eMSC-FLT3L), n=9 (eMSC-Ctrl+poly(I:C), eMSC-FLT3L+poly(I:C)) mice per group for the MFI, two independent experiments, one-way ANOVA-test with Tukey's multiple comparisons. Box plots show median, 25th–75th percentiles, minimum–maximum whiskers, with all data points displayed. Source data are provided as a Source Data file.

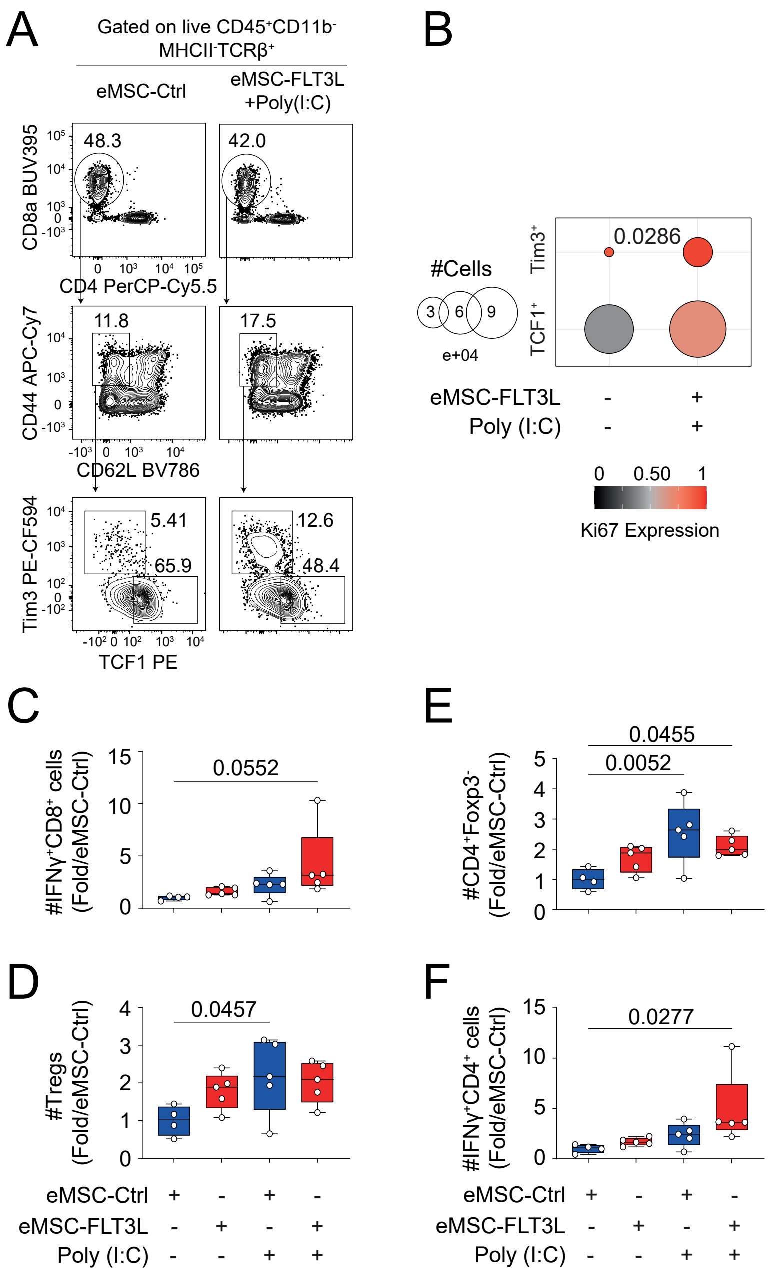

<u>Supplementary Figure 4:</u> Intratumoral delivery of eMSC-FLT3L + poly(I:C) increase proliferative stem-like CD8$^+$ T cells, IFNγ$^+$CD8$^+$ and IFNγ$^+$CD4$^+$ but not T regulatory cells.

(A) Representative flow cytometry plots of CD8 T cells in the tumor-draining lymph node at day 15.

(B) Quantification of the absolute number and Ki67 expression of exhausted (Tim3$^+$) and stem-like (TCF1$^+$) T cells. Dots represent the absolute number of cells while the colors represent the expression level of Ki67. n=4 mice per group, one experiment, statistics are done on the absolute numbers, two-tailed Mann-Whitney test.

(C-F) Quantification of the absolute number of IFNγ$^+$CD8$^+$ T cells (C), CD4$^+$Foxp3$^+$ T regulatory cells (Tregs) (D), CD4$^+$Foxp3$^-$ cells (E) and IFNγ$^+$CD4$^+$ cells (F) at day 15. n=4 (eMSC-Ctrl), n=5 (other groups) mice per group, one experiment, one-way ANOVA-test with Dunnett's multiple comparison test. Box plots show median, 25th–75th percentiles, minimum–maximum whiskers, with all data points displayed.

(B-F) Source data are provided as a Source Data file.

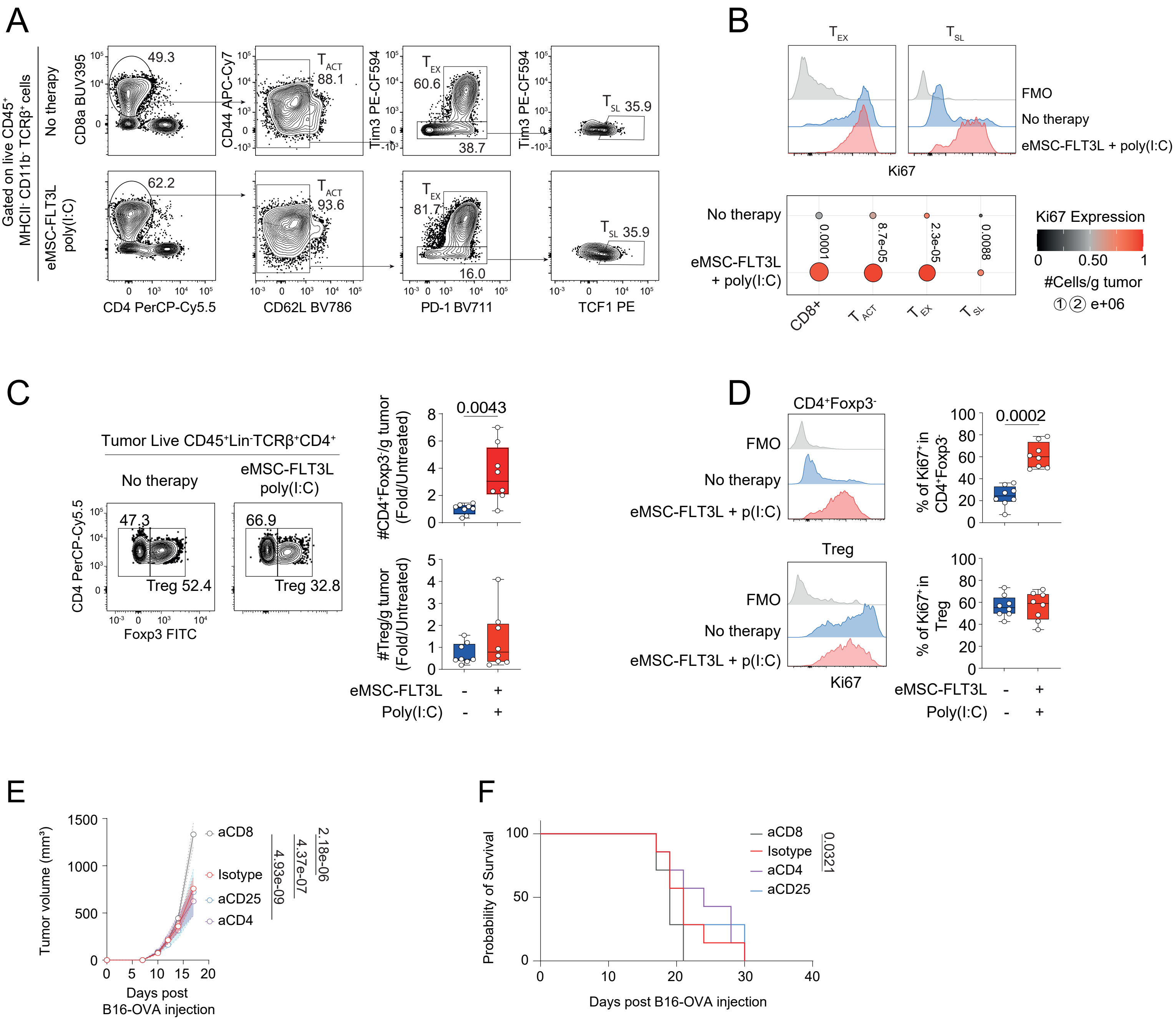

A
Gated on live CD45+ MHCII- CD11b- TCRβ+ cells
No therapy
eMSC-FLT3L poly(I:C)
CD8a BUV395
CD4 PerCP-Cy5.5
49.3
62.2
CD44 APC-Cy7
CD62L BV786
TACT 88.1
TACT 93.6
Tim3 PE-CF594
PD-1 BV711
TEX 60.6
38.7
TEX 81.7
16.0
TCF1 PE
TSL 35.9
TSL 35.9
B
TEX
TSL
FMO
No therapy
eMSC-FLT3L + poly(I:C)
Ki67
No therapy
eMSC-FLT3L + poly(I:C)
0.0001
8.7e-05
2.3e-05
0.0088
CD8+
TACT
TEX
TSL
Ki67 Expression
0
0.50
1
#Cells/g tumor
① ② e+06
C
Tumor Live CD45+Lin-TCRβ+CD4+
No therapy
eMSC-FLT3L poly(I:C)
CD4 PerCP-Cy5.5
Foxp3 FITC
47.3
Treg 52.4
66.9
Treg 32.8
#CD4+Foxp3-/g tumor (Fold/Untreated)
0.0043
#Treg/g tumor (Fold/Untreated)
eMSC-FLT3L - +
Poly(I:C) - +
D
CD4+Foxp3-
FMO
No therapy
eMSC-FLT3L + p(I:C)
Treg
Ki67
% of Ki67+ in CD4+Foxp3-
0.0002
% of Ki67+ in Treg
eMSC-FLT3L - +
Poly(I:C) - +
E
Tumor volume (mm3)
Days post B16-OVA injection
aCD8
Isotype
aCD25
aCD4
2.18e-06
4.37e-07
4.93e-09
F
Probability of Survival
Days post B16-OVA injection
aCD8
Isotype
aCD4
aCD25
0.0321

<u>Supplementary Figure 5:</u> Intratumoral delivery of eMSC-FLT3L + poly(I:C) stimulates infiltration and proliferation of CD4+ T cells but not of T regulatory cells.

(A-B) Representative flow cytometry plots (A) and quantification (B) of activated (T_{ACT}), exhausted (T_{EX}), and stem-like (T_{SL}) $CD8^+$ T cells. Dots represent the absolute number of cells/g tumor while the colors represent the expression level of Ki67. n=4 mice per group, one experiment, statistics are done on the absolute numbers, two-tailed unpaired t-test.

(C) Representative flow cytometry plots and absolute number quantification of $CD4^+Foxp3^-$ and Treg. Results are shown as fold change to control (Untreated)**.** n=8 mice per group, two independent experiments, two-tailed unpaired t-test.

(D) Representative flow cytometry plots and frequency of $Ki67^+$ cells in $CD4^+Foxp3^-$ cells and Treg**.** n=8 mice per group, two independent experiments, two-tailed unpaired t-test.

(C-D) Box plots show median, 25th–75th percentiles, minimum–maximum whiskers, with all data points displayed.

(E) Tumor growth curves. n=7 mice per group, one experiment, two-way ANOVA-test with Tukey's multiple comparisons. A line represents the mean and SEM is shown with the colored area.

(F) Survival curves. n=7 mice per group, one experiment, log-rank (Mantel-Cox) test.

(B-F) Source data are provided as a Source Data file.

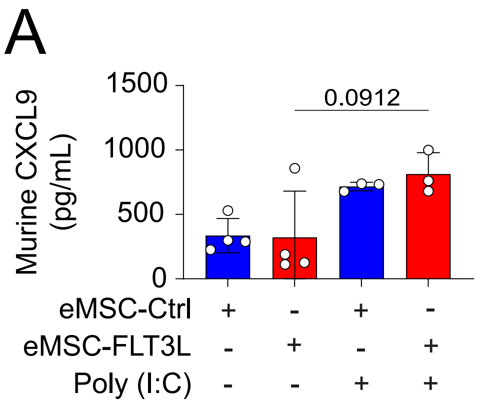

Supplementary Figure 6: poly(I:C) monotherapy is sufficient to upregulate CXCL9 expression in tumor homogenates.

(**A**) Murine CXCL9 ELISA done on tumor homogenates 24h after poly(I:C) injection. n=3 (eMSC-Ctrl+poly(I:C), eMSC-FLT3L+poly(I:C)), n=4 (eMSC-Ctrl, eMSC-FLT3L), one experiment, Kruskal-Wallis test with Dunn's multiple comparisons. Data are presented as mean values ± SD. Source data are provided as a Source Data file.

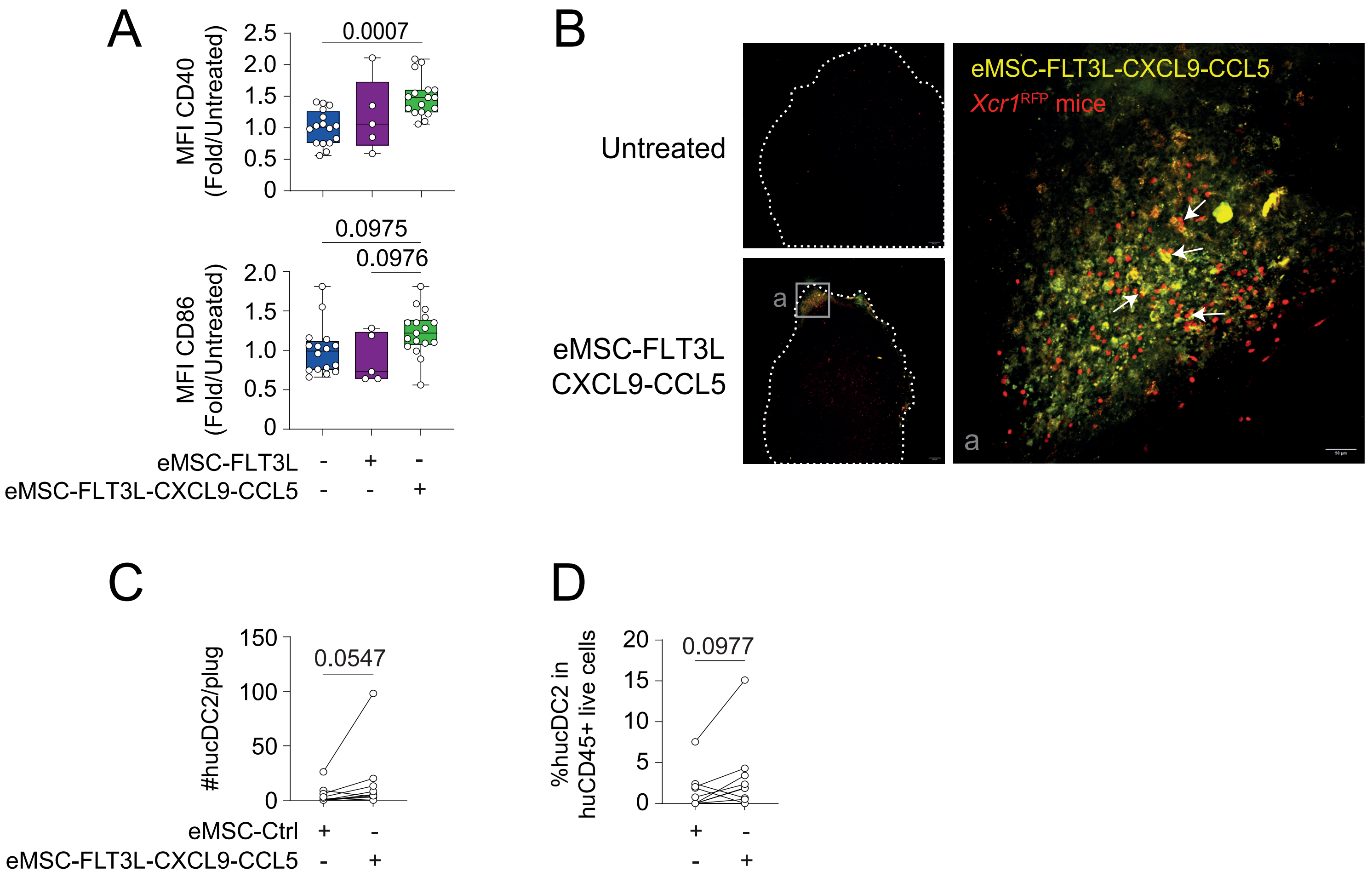

<u>Supplementary Figure 7: eMSC-FLT3L-CXCL9-CCL5 are localized at the border of the tumor and attract hucDC2s in synthetic niches in reconstituted BRGSF mice.</u>

(A) Quantification by flow cytometry of the mean fluorescence intensity (MFI) of CD40 and CD86 at day 17. Results are shown as fold change to control (Untreated). n=16 (untreated), n=17 (eMSC-FLT3L-CXCL9-CCL5), n=5 (eMSC-FLT3L) mice per group, three independent experiments, one-way ANOVA with Tukey's multiple comparisons test. Box plots show median, 25th–75th percentiles, minimum–maximum whiskers, with all data points displayed.

(**B**) Immunofluorescence imaging of YUMM-OVA tumors injected in *Xcr1*^{RFP} mice. These tumors received 3 injections of DMEM (Untreated) or eMSC-FLT3L-CXCL9-CCL5. Images were taken with a spinning disk 24h after the last injection. cDC1s are in red, while eMSC-FLT3L-CXCL9-CCL5 are in yellow. Tumors are delimited with the doted white lines. Representative of three mice, two independent experiments.

(**C-D**) Quantification of the absolute number (C) and frequencies (D) of human cDC2/plug. n=16 plugs per group, three independent experiments, two-tailed Wilcoxon matched-pairs signed rank test.

(A, C-D) Source data are provided as a Source Data file.

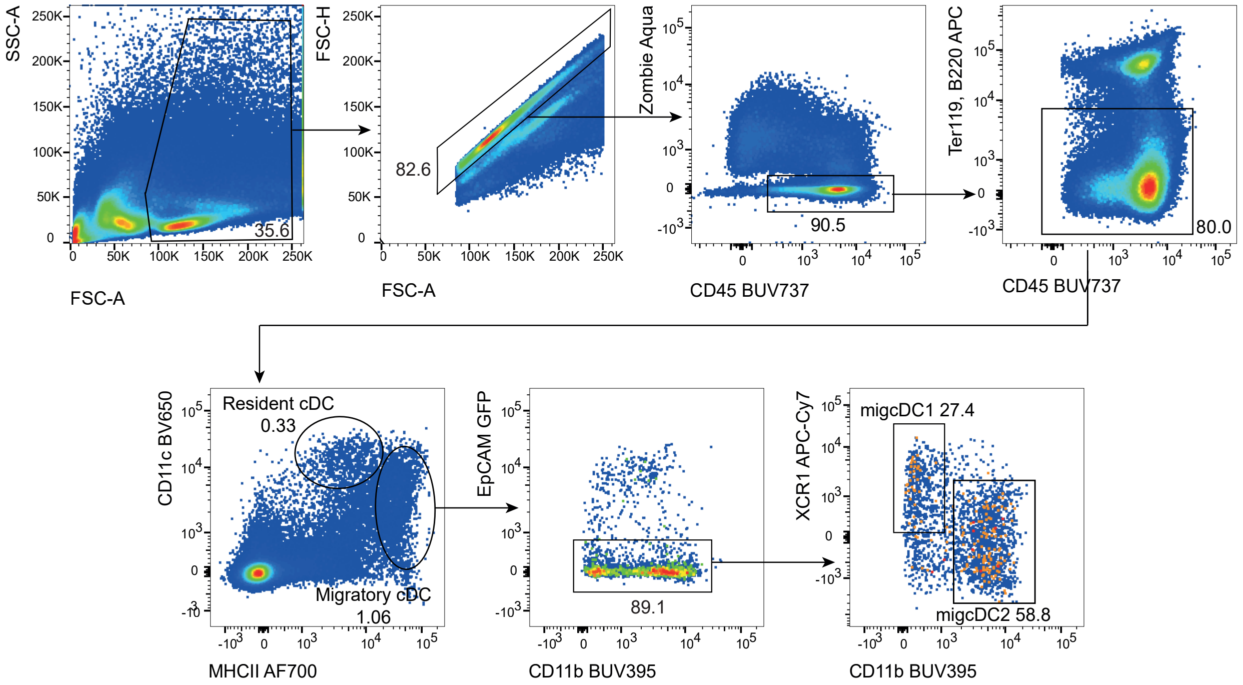

<u>Supplementary Figure 8: Gating strategy used to identify migratory cDC1s and cDC2s within tumor draining lymph nodes.</u>

(A) Gating strategy used to identify migratory cDC1s and cDC2s within tumor draining lymph nodes in Figure 1L and 4C.

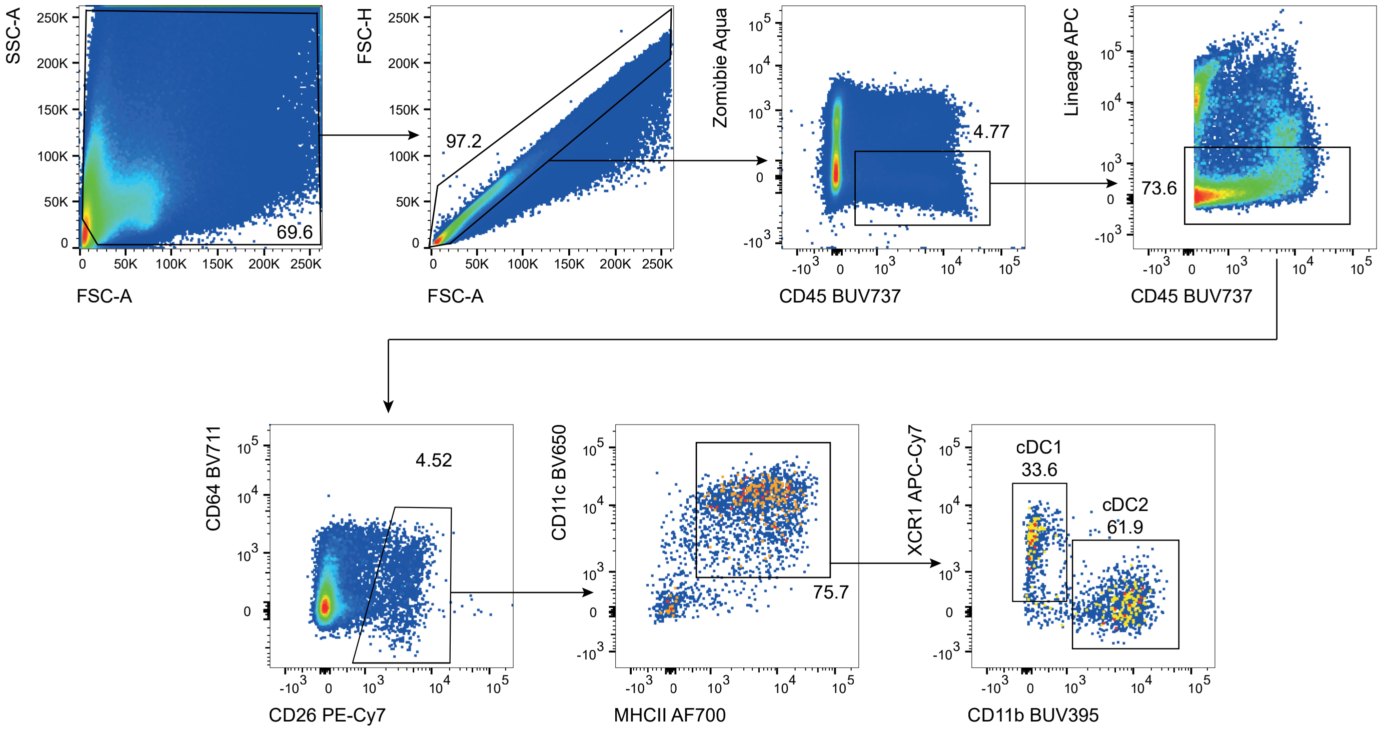

Supplementary Figure 9: Gating strategy used to identify cDC1s and cDC2s within tumors.

(A) Gating strategy used to identify cDC1s and cDC2s within tumors in Figure 1K, 3B-C, 6B-C, 6I, 7G-H and 7L. Lineage: Ter119, CD3, NK1.1, Ly6G, SiglecF, B220.

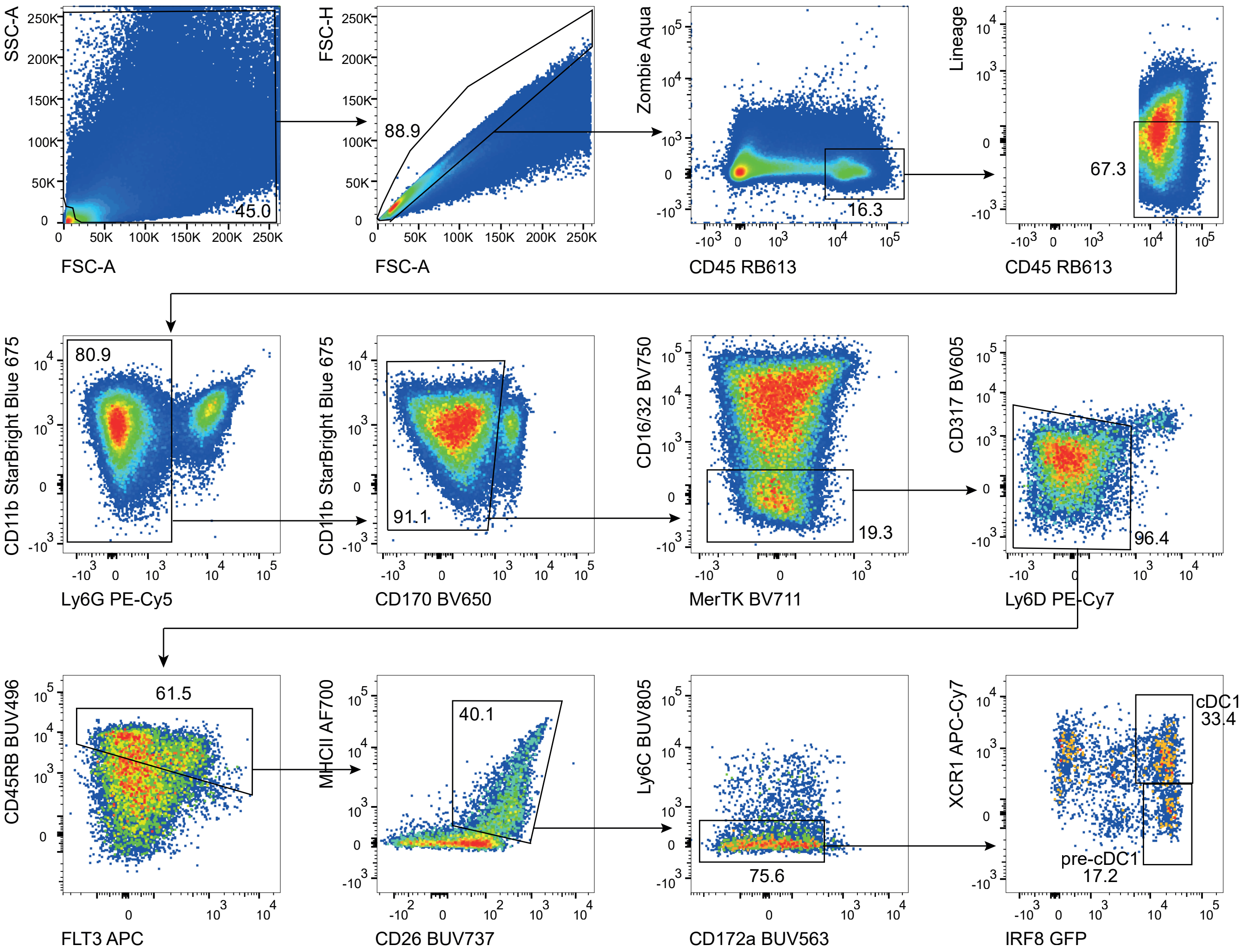

<u>Supplementary Figure 10: Gating strategy used to identify pre-cDC1s within tumors.</u>

(A) Gating strategy used to identify pre-cDC1s within tumors in Figure 7E. Lineage: Ter119, CD3, NK1.1, CD19.

Supplementary Table 1: **Resource table**.

REAGENT or RESOURCE	SOURCE	IDENTIFIER	DILUTION
**Mouse antibodies**			
CD11b AF700 (M1/70)	Biolegend	Cat# 101222, RRID:AB_493705	200
CD11b BV421 (M1/70)	Biolegend	Cat# 101236, RRID:AB_11203704	200
CD11b FITC (M1/70)	Biolegend	Cat# 101206, RRID:AB_312789	200
CD11b BUV395 (M1/70)	BD	Cat# 565976, RRID:AB_2721166	200
CD11b BV785 (M1/70)	Biolegend	Cat# 101243, RRID:AB_2561373	200
CD11b StarBright Blue 675 (5C.6)	Bio-Rad	Cat#MCA711SBB675, RRID:AB_3101464	200
CD11c PeCy7 (N418)	Biolegend	Cat# 117318, RRID:AB_493568	200
CD11c PE/Dazzle™ 594 (N418)	Biolegend	Cat# 117348, RRID:AB_2563655	200
CD16/32 BV510 (93)	Biolegend	Cat# 101333, RRID:AB_2563692	200
CD24 BV510 (M1/69)	BioLegend	Cat# 101831, RRID: AB_2563894	400
CD26 PeCy7 (H194-112)	Biolegend	Cat# 137810, RRID:AB_2564312	200
CD3e PE (145-2c11)	Biolegend	Cat# 100308, RRID:AB_312673	100
CD4 BV510 (RM4-5)	Biolegend	Cat# 100559, RRID:AB_2562608	400
CD4 PercpCy5.5 (RM4-5)	Biolegend	Cat# 100540, RRID:AB_893326	400
CD4 AF700 (RM4-5)	Biolegend	Cat# 100536, RRID:AB_493701	400
CD44 APC/Fire750 (IM7)	Biolegend	Cat# 103062, RRID:AB_2616727	200
CD45 BUV395 (30-F11)	eBioscience	Cat# 363-0451-82, RRID:AB_2925264	800

CD45 BUV737 (30-F11)	eBioscience	Cat# 367-0451-82, RRID: AB_2895963	800
CD45 APC/Fire750 (30F11)	Biolegend	Cat# 103154, RRID:AB_2572116	800
CD45 RB613 (I3/2)	BD	Cat # 758191, RRID:AB_3690340	400
CD45 AF700 (30-F11)	Biolegend	Car# 103128, RRID:AB_493715	800
CD45 BUV805 (30F11)	BD	Cat# 568336, RRID:AB_3684191	800
CD45.1 PE-Cy7 (A20)	Biolegend	Cat# 110729, RRID:AB_1134170	600
CD45.1 BV605 (A20)	Biolegend	Cat# 110738, RRID:AB_2562565	600
CD45.2 PerCp-Cy5 (104)	eBioscience	Cat# 45-0454-82 RRID: AB_953590	200
CD45RB APC (C363-16A)	BioLegend	Cat# 103319; RRID: AB_2565228	200
CD49a AF647 (Ha31/8)	BD	Cat# 562113, RRID:AB_11153312	200
CD62L FITC (MEL-14)	Biolegend	Cat# 104406, RRID:AB_313093	200
CD62L BV785 (MEL-14)	Biolegend	Cat# 104440, RRID:AB_2629685	200
CD64 BV711 (X54-5/7.1)	Biolegend	Cat# 139311, RRID:AB_2563846	200
CD80 BV421 (16-10A1)	Biolegend	Cat# 104725, RRID:AB_10900989	400
CD8a (KT15) FITC	BioRad	Cat# MCA609G, RRID: AB_321407	400
CD8a BV785 (53-6.7)	Biolegend	Cat# 100750, RRID:AB_2562610	400
CD8b BUV395 (H35-17.2)	BD	Cat# 740278, RRID:AB_2740017	400
CX3CR1 BV785 (SA011F11)	Biolegend	Cat# 149029, RRID:AB_2565938	200
Ki67 APC (SolA15)	Invitrogen	Cat# 56-5698-82, RRID:AB_2637480	200

Ly6C APC/ Fire750 (HK1.4)	Biolegend	Cat# 128046, RRID:AB_2616731	200
Ly6C BUV805 (HK1.4)	BD	Cat# 755202, RRID:AB_3687609	200
Ly6G BV650 (1A8)	Biolegend	Cat# 127641, RRID:AB_2565881	400
I-A/I-E APC (M5/114.15.2)	Biolegend	Cat# 107614, RRID:AB_313329	400
I-A/I-E AF700 (M5/114.15.2)	Biolegend	Cat# 107622, RRID:AB_493727	400
PD-1 (CD279) BV711 (29F.1A12)	Biolegend	Cat# 135231, RRID:AB_2566158	200
SiglecH BUV395	BD	Cat# 747669, RRID: AB_2744230	200
SiglecF BV421 (E50-2440)	BD	Cat# 562681, RRID:AB_2722581	400
TCRb BV650 (H57-597)	Biolegend	Cat# 109251, RRID:AB_2810348	400
Tim3 PE (RMT3-23)	BD	Cat# 568428; RRID: AB_345377	200
PD-L1 BV605	Biolegend	Cat# 124321, RRID:AB_2563635	400
XCR1 PE (ZET)	Biolegend	Cat# 148204, RRID:AB_2563843	200
XCR1 BV785 (ZET)	Biolegend	Cat# 148225, RRID:AB_2783119	200
NK1.1 PE-Cy7 (clone S17016D)	Biolegend	Cat# 156513; RRID:AB_2888852	200
CD40 PE-Dazzle584 (3/23)	Biolegend	Cat# 124629; RRID: AB_2572184	200
CD40 RY610 (3/23)	BD	Cat# 759052, RRID:AB_3691150	200
CD86 FITC (GL-1)	Biolegend	Cat# 105005, RRID: AB_313148	200
CD86 PE (GL-1)	Biolegend	Cat# 105008, RRID:AB_313151	200
CD86 AF700 (GL-1)	Biolegend	Cat#105023, RRID:AB_493720	200
NKp46 BV421 (29A1.4)	Biolegend	Cat# 137612, RRID:AB_2563104	200
EpCAM BV510 (G8.8)	Biolegend	Cat# 101831; RRID: AB_2563894	400
Podoplanin/gp38 BV421 (8.1.1)	Biolegend	Cat#127423 ; RRID: AB_2814017	200

CD31 BV785 (390)	Biolegend	Cat#102435 ; RRID: AB_2810334	400
ICAM-1 PE (YN1/1.7.4)	Biolegend	Cat#116107 ; RRID: AB_313698	400
IFNγ BV785 (XMG1)	Biolegend	Cat# 505837; RRID: AB_2629667	100
IFNγ BV650 (XMG1)	Biolegend	Cat# 505831; RRID: AB_11142685	100
TCF1/TCF7 PE (S33-966)	BD	Cat# 564217; RRID: AB_2687845	100
Foxp3 FITC (FJK-16S)	eBioscience	Cat# 11-5773-82 ; RRID: AB_465243	200
CD172a PE-Cy7 (P84)	Biolegend	Cat# 144007; RRID: AB_2563545	200
CD172a APC (P84)	BD	Cat# 560106, RRID:AB_1645218	200
Streptavidin APC-R700	BD	Cat# 565144, RRID:AB_2869657	800
FLT3 PE (A2F10)	eBioscience	Cat# 12-1351-82, RRID: AB_465859	200
ESAM PE (1G8/ESAM)	Biolegend	Cat# 136203; RRID: AB_1953300	200
TNFα PE-Cy7 (MP6-XT22)	Biolegend	Cat# 506305; RRID: AB_315426	100
IL2 PE-Cy5 (JES6-5H)	Biolegend	Cat# 503824; RRID: AB_2123674	100
Granzyme B FITC (QA16A02)	ebioscience	Cat# 11-8898-82; RRID: AB_10733414	100
CD4 PerCP-Cy5.5 (GK1.5)	Biolegend	Cat# 100434; RRID: AB_893324	400
CD73 PE (eBioTY/11.8)	Biolegend	Cat# 127205; RRID: AB_1089065	100
CD90.2 APC (30–H12)	Biolegend	Cat# 105311; RRID: AB_313182	100
CD140a APC (APA5)	Biolegend	Cat# 135908; RRID: AB_2043970	100
CCR5 APC (HM-CCR5)	Biolegend	Cat# 107011; RRID: AB_2074528	100

CXCR3 (S18001A) PE	Biolegend	Cat# 155903; RRID: AB_2783130	100
Anti-mouse CD28, (37.51), Ultra-LEAF™	Biolegend	Cat# 102116; RRID: AB_11147170	2000
Anti-mouse CD3e (17A2)	Biolegend	Cat# 100243; RRIB: AB_2563946	200
Anti-mouse IL-4 (clone 11B11) Ultra-LEAF™	Biolegend	Cat# 504122, RRID: AB_11149679	200
InVivoMAb rat IgG2b isotype control, anti-keyhole limpet hemocyanin (clone LTF-2)	BioXcell	Cat# BE0090; RRID: AB_1107780	See Methods section
InVivoMAb anti-mouse agonist PD-1 (29F.1A12)	BioXcell	Cat# BE0273; RRID: AB_2687796 Lot: 883023J1	See Methods section
InVivoMAb anti-mouse agonist CTLA-4 (CD152, Clone 9H10)	BioXcell	Cat# BE0131; RRID: AB_10950184 Lot: 834323S1	See Methods section
InVivoMab anti-mouse CD4 (GK1.5)	BioXcell	Cat# BE0003-1; RRID: AB_1107636 Lot: 805422A1	See Methods section
InVivoMab anti-mouse CD8 (2.43)	BioXcell	Cat# BE0061; RRID: AB_1125541 Lot: 811522A2	See Methods section
InVivoMab anti-mouse NK1.1 (PK136)	BioXcell	Cat# BE0036; RRID: AB_1107737 Lot: 796521N1	See Methods section
InVivoMAb anti-mouse CD25 (PC-61.5.3)	BioXcell	Cat# BE0012; RRID:AB_1107619 Lot:795321D1	See Methods section

InVivoMAb anti-mouse CD40 (FGK4.5)	BioXcell	Cat# BE0016-2; RRID:AB_1107647 Lot: 805122F1	See Methods section
CD45 Biotin (30-F11)	Biolegend	Cat# 103103; RRID:AB_312968	400
Ly6G Biotin (1A8)	Biolegend	Cat# 127604; RRID: AB_1186105	400
CD3 Biotin (145-2C11)	Biolegend	Cat# 100304; RRID: AB_312669	400
CD19 Biotin (6D5)	Biolegend	Cat# 115503; RRID: AB_313638	400
CD45R/B220 Biotin (RA3-6B2)	Biolegend	Cat# 103203; RRID: AB_312988	400
NK1.1 Biotin (PK136)	Biolegend	Cat# 108703; RRID: AB_313390	400
SiglecF Biotin (F17007L)	Biolegend	Cat# 155512; RRID: AB_2814066	400
TER119 Biotin (TER-119)	Biolegend	Cat# 116203; RRID: AB_313704	400
CD11b Biotin (M1/70)	Biolegend	Cat# 101204, RRID:AB_312787	400
I-A/I-E Biotin (M5/114.15.2)	Biolegend	Cat# 107604, RRID:AB_313319	400
**Human antibodies**			
Human FLT3L Biotin (Polyclonal)	Bio-Techne/R&D systems	Cat# BAF308; RRID:AB_2278494	100
NKp46 Biotin (9E2)	Biolegend	Cat#331906, RRID:AB_1027671	40
CD3 Biotin (OKT3)	Biolegend	Cat#317319, RRID:AB_10918432	400
CD19 Biotin (HIB19)	Biolegend	Cat#302203, RRID:AB_314233	400
CD56 Biotin (HCD56)	Biolegend	Cat#318319, RRID:AB_893392	40
CD66b Biotin (G10F5)	Biolegend	Cat#305120, RRID:AB_2566608	40
CD203c Biotin (REA826)	Miltenyi	Cat#130-112-811, RRID:AB_2656166	100
CD20 Biotin (2H7)	Biolegend	Cat#302349, RRID:AB_2565523	200
HLA-DR BUV737 (L243)	BD	Cat#753688, RRID:AB_3687372	100

CD163 BUV661 (GHI/61)	BD	Cat#741645, RRID:AB_2871044	100
BDCA-2 BUV615 (V24-785)	BD	Cat#751078, RRID:AB_2875114	100
CD16 BUV395 (3G8)	BD	Cat#563785	100
BTLA BV421 (MIH26)	Biolegend	Cat#344511, RRID:AB_2566507	
CD5 BV605 (L17F12)	Biolegend	Cat#364019, RRID:AB_2565940	100
CD123 BV650 (6H6)	Biolegend	Cat#306019, RRID:AB_11218792	100
CD141 BV711 (M80)	Biolegend	Cat#344135, RRID:AB_3097423	40
CD14 BV786 (M5E2)	BD	Cat#563699	100
AXL FITC (108724)	RnD Systems	Cat#18624513	100
XCR1 PerCP-Cy5.5 (S15046E)	Biolegend	Cat#372629, RRID:AB_2924564	100
CLEC10A PE (H037G3)	Biolegend	Cat#354703, RRID:AB_11219202	100
CD88 PE-DAZZLE (S5/1)	Biolegend	Cat#344317, RRID:AB_2750446	100
CD45 PE-Cy5 (HI30)	BD	Cat#555484, RRID:AB_395876	200
CD1c PE-Cy7 (L161)	Biolegend	Cat#331515, RRID:AB_1953227	100
CLEC9A APC (8F9)	Biolegend	Cat#353805, RRID:AB_2565518	100
CX3CR1 R718 (2A9-1)	BD	Cat#752200, RRID:AB_2917307	100
CD45RA APC-Cy7 (HI100)	Biolegend	Cat#304127, RRID:AB_10708419	100
**Chemicals, peptides, and recombinant proteins**			
Collagenase D	Sigma	Cat# 11088866001	
Collagenase A	Sigma	Cat# 10103586001	
DNase I	Sigma	Cat# 10104159001	
Dispase II	Sigma	Cat# D4693	
HEPES	Gibco	Cat# 11560496	
Penicillin/Streptomycin	Gibco	Cat# 11528876	

Bovine Serum Albumin Raction V (BSA)	Euromedex	Cat# 04-100-812	
Accucheck counting beads	ThermoFischer	Cat# PCB100	
DMEM Medium, GlutaMAX™ Supplement	Gibco	Cat# 10566024	
RPMI 1640 Medium, GlutaMAX™ Supplement	Gibco	Cat# 61870036	
HBSS, calcium, magnesium	Gibco	Cat# 24020117	
Trypsin-EDTA (0.25%), phenol red	Gibco	Cat# 25200056	
2-mercaptoéthanol	Gibco	Cat# 11528926	
Fetal Bovine Serum, qualified, Mexico	Gibco	Cat# 12676029	
ACK Lysing Buffer	Gibco	Cat# A1049201	
Ovalbumin peptide SIINFEKL (H-2 Kb)	IVA Lifesciences	Cat# 6-7015-901	
iTAg Tetramer/PE – H-2 Kb OVA (SIINFEKL)	MBL	Cat# TB-5001-1	20
Recombinant mouse IL12p70 (carrier-free)	Biolegend	Cat# 577004	
Zombie Yellow™ Fixable	Biolegend	Cat#423104	1000
Zombie NIR™ Fixable	Biolegend	Cat#423106	1000
Zombie Aqua™ Fixable	Biolegend	Cat#423102	1000
Live/Dead Fixable Blue	ThermoFisher	Cat#L23105	1000

7-AAD	Biolegend	Cat#420404	100
Puromycin	Gibco	Cat#A1113803	
DAPI (4',6-Diamidino-2-Phenylindole, Dilactate)	Biolegend	Cat# 422801	1000
AMG 487 Antagonist CXCR3	MedChemExpress	Cat# HY-15319	
Maraviroc Antagonist CCR5	MedChemExpress	Cat# HY-13004	
Recombinant mouse IL-2 (carrier-free)	Biolegend	Cat# 575402	
Geltrex™ LDEV-Free Reduced Growth Factor Basement Membrane Matrix	Gibco	Cat# A1413201	
PMA (Phorbol 12-myristate 13-acetate)	Sigma-Aldrich	Cat# 16561-29-8	
Ionomycin	Sigma-Aldrich	Cat# 56092-82-1	
Recombinant human Flt3-L	Amgen Inc.	Cat# CDX-301	
Nano-Glo® Fluorofurimazine In Vivo Substrate	Promega	Cat# N4110	
Alanine Aminotransferase (ALT/GPT) Activity Assay Kit	ThermoFisher	Cat# EEA001	
Polybrene	Santa Cruz Biotechnology	Cat# sc-134220	
4% paraformaldehyde solution	ThermoFisher Scientific	Cat# J19943.K2	
**Plasmid and vectors**			
pMX-IRES-GFP	Origene	NM_001204502.1 (*1*)	

pMX-IRES-Cherry	Origene	NM_000460.2 (*1*)	
pMX-huFLT3L-IRES-GFP	Dr. Guermonprez	(*1*)	
pMX-huFLT3L-IRES-mCherry	This paper		
Human CXCL9 Sequence	Cat#: MHS6278-202806039	Dharmacon horizon	
Human CCL5 Sequence	Cat#: MHS6278-202757576	Dharmacon horizon	
pMX-huCXCL9-IRES-GFP	This paper		
pMX-huCCL5-IRES-GFP	This paper		
pMX-huCCL5-P2A-huCXCL9-IRES-GFP	This paper	GeneArt Gene Synthesis	
NanoLuc lentiviral vector	Dr. Laleh Majlessi	N/A	
**Experimental models: Cell lines**			
B16F10		(*2*)	
B16-huFLT3L-GFP		(*2*)	
B16-OVA	This paper		
YUMM-OVA	This paper		
E0771	Dr. Stéphanie Hugues	ATCC Number: CRL-3461	
TC-1-Luc	Dr. Alexandre Boissonnas	N/A	
MC38	Dr. Philippe Bousso	Cat# SCC172 (Sigma-Aldrich)	
Mesenchymal stromal cells (MSCs)	Dr. Loredana Saveanu	N/A	
eMSC-GFP	This paper		

eMSC-mCherry	This paper		
eMSC-GFP-mCherry	This paper		
eMSC-huFLT3L-GFP	This paper		
eMSC-huFLT3L-mCherry	This paper		
eMSC-huCCL5-GFP	This paper		
eMSC-huCXCL9-GFP	This paper		
eMSC-huFLT3L-mCherry-huCXCL9-P2A-huCCL5-GFP	This paper		
eMSC-NanoLuc	This paper		
**Critical commercial assays**			
Human Flt-3 Ligand/FLT3L Quantikine ELISA Kit	R&D	Cat# DFK00	
LEGENDplex™ Mouse Proinflammatory Chemokine Panel (13-plex) with V-bottom Plate	Biolegend	Cat# 740451	
LEGENDplex™ MU Proinflam. Chemokine Panel 2 (8-plex)	Biolegend	Cat# 741068	
Cytofix/cytoperm Kit	BD Bioscience	Cat# 554714	
Transcription Factor Staining Buffer Set	ThermoFischer	Cat# 00-5523-00	
EasySep™ Mouse Hematopoietic Progenitor Cell Isolation Kit	Stemcell	Cat# 19856	
Human CXCL9 ELISA	R&D Sytstems	Cat# DY392-05	
Human CCL5 ELISA	R&D Sytstems	Cat# DY278-05	

Corning® Transwell® polycarbonate, ø inserts 6.5 mm, porosity 5 µm (12 inserts in 24-well plate)	Sigma-Aldrich Corning	Cat# 003421	
**Experimental models: Organisms/strains**			
mouse: C57BL/6J	Janvier Labs	RRID:IMSR_JAX:000664	
mouse: CD45.1 (CByJ.SJL(B6) *Ptprc*^{a}/J)	Janvier Labs	RRID:IMSR_JAX:006584	
mouse: OT-1 Rag2-/- CD45.1	Dr. Sebastian Amigorena	(*3*)	
mouse: Rosa-DTA (B6.129P2 *Gt(ROSA)26Sor*$^{tm1(DTA)Lky}$/J)	Dr. Marc Dalod	RRID:IMSR_JAX:009669	
mouse: ROSA26-LSL-RFP (B6.Cg *Gt(ROSA)26Sor*^{tm1Hjf}*/J*)	Dr. Marc Dalod	RRID:IMSR_JAX:038164	
mouse: *Xcr1-Cre* (B6 *Xcr1*^{tm1Ciphe})	Dr. Marc Dalod	(*4*)	
Mouse: ROSA26-LSL-tdTomato (B6.Cg *Gt(ROSA)26Sor*$^{tm14(CAG\text{-}tdTomato)Hze}$/J)	Dr. Tessa Bergsbaken	RRID:IMSR JAX:007914	
mouse: *Irf8*-GFP (B6.Cg *Irf8*$^{tm2.1Hm}$/J)	The Jackson Laboratory	RRID: IMSR_JAX:027084	
mouse: BALB/c *Rag2*$^{-/-}$ *IL2rγ*$^{-/-}$ *Sirpα*^{NOD} (BRGS), BALB/c	Human Disease Model Core facility -	(*5*)	

Rag2$^{-/-}$ *IL2rγ*$^{-/-}$ *Sirpα*^{NOD} *Flt3*$^{+/+}$	Pasteur Institute		
mouse: BALB/c *Rag2*$^{-/-}$ *IL2rγ*$^{-/-}$ *Sirpα*^{NOD} (BRGS), BALB/c *Rag2*$^{-/-}$ *IL2rγ*$^{-/-}$ *Sirpα*^{NOD} *Flt3*$^{-/-}$	Human Disease Model Core facility - Pasteur Institute	(*5*)	
**Software and algorithms**			
BD FACSDiva	BD		
FlowJo v10.10	BD	https://www.flowjo.com	
GraphPad Prism 10	GraphPad Software	https://www.graphpad.com	
RStudio 4.4.2	The R Foundation	https://www.r-project.org	
Morpheus	Broad institute	https://software.broadinstitute.org/morpheus/	
LEGENDplex™ Data Analysis Software	Biolegend	https://legendplex.qognit.com/	
NIS-Elements AR 6. 10	Nikon		
Image J v1. 54f	(*6*)	https://imagej.nih.gov/ij/	
QuPath 0.4.4	Qupath software	https://qupath.github.io/	
ID7000 Software 2.2.1.17271	Sony		
Living Image 4.8.2 Software	Caliper Life Sciences		
Gen5 3.12 software	Agilent BioTek		

References

1. G. Anselmi, K. Vaivode, C.-A. Dutertre, P. Bourdely, Y. Missolo-Koussou, E. Newell, O. Hickman, K. Wood, A. Saxena, J. Helft, F. Ginhoux, P. Guermonprez, Engineered niches support the development of human dendritic cells in humanized mice. *Nat Commun* **11**, 2054 (2020).

2. P. Bourdely, G. Anselmi, K. Vaivode, R. N. Ramos, Y. Missolo-Koussou, S. Hidalgo, J. Tosselo, N. Nuñez, W. Richer, A. Vincent-Salomon, A. Saxena, K. Wood, A. Lladser, E. Piaggio, J. Helft, P. Guermonprez, Transcriptional and Functional Analysis of CD1c+ Human Dendritic Cells Identifies a CD163+ Subset Priming CD8+CD103+ T Cells. *Immunity* **53**, 335-352.e8 (2020).

3. L. L. Niborski, P. Gueguen, M. Ye, A. Thiolat, R. N. Ramos, P. Caudana, J. Denizeau, L. Colombeau, R. Rodriguez, C. Goudot, J.-M. Luccarini, A. Soudé, B. Bournique, P. Broqua, L. Pace, S. Baulande, C. Sedlik, J.-P. Quivy, G. Almouzni, J. L. Cohen, E. Zueva, J. J. Waterfall, S. Amigorena, E. Piaggio, CD8+T cell responsiveness to anti-PD-1 is epigenetically regulated by Suv39h1 in melanomas. *Nat Commun* **13**, 3739 (2022).

4. R. Mattiuz, C. Wohn, S. Ghilas, M. Ambrosini, Y. O. Alexandre, C. Sanchez, A. Fries, T.-P. Vu Manh, B. Malissen, M. Dalod, K. Crozat, Novel Cre-Expressing Mouse Strains Permitting to Selectively Track and Edit Type 1 Conventional Dendritic Cells Facilitate Disentangling Their Complexity in vivo. *Front. Immunol.* **9** (2018).

5. G. Masse-Ranson, M. Dusséaux, O. Fiquet, S. Darche, M. Boussand, Y. Li, S. Lopez-Lastra, N. Legrand, E. Corcuff, A. Toubert, M. Centlivre, T. Bruel, H. Spits, O. Schwartz, Y. Lévy, H. Strick-Marchand, J. P. Di Santo, Accelerated thymopoiesis and improved T-cell responses in HLA-A2/-DR2 transgenic BRGS-based human immune system mice. *Eur J Immunol* **49**, 954–965 (2019).

6. C. A. Schneider, W. S. Rasband, K. W. Eliceiri, NIH Image to ImageJ: 25 years of image analysis. *Nat Methods* **9**, 671–675 (2012).
