# Supplementary material for: Intratumoral delivery of FLT3L with CXCR3/CCR5 ligands promotes XCR1+ cDC1 infiltration and activates anti-tumor immunity
Source: Nat Commun. 2025 Dec 30;17:1258. doi: 10.1038/s41467-025-68018-3 (PMC12865026; doi:10.1038/s41467-025-68018-3)
Supplement: Supplementary file 2 — Reporting Summary [file 41467_2025_68018_MOESM2_ESM.pdf]

nature portfolio

Corresponding author(s): Pierre Guermonprez

Last updated by author(s): 13/12/2025

Reporting Summary

Nature Portfolio wishes to improve the reproducibility of the work that we publish. This form provides structure for consistency and transparency in reporting. For further information on Nature Portfolio policies, see our Editorial Policies and the Editorial Policy Checklist.

Statistics

For all statistical analyses, confirm that the following items are present in the figure legend, table legend, main text, or Methods section.

n/a	Confirmed	
☐	☒	The exact sample size (*n*) for each experimental group/condition, given as a discrete number and unit of measurement
☐	☒	A statement on whether measurements were taken from distinct samples or whether the same sample was measured repeatedly
☐	☒	The statistical test(s) used AND whether they are one- or two-sided<br>*Only common tests should be described solely by name; describe more complex techniques in the Methods section.*
☒	☐	A description of all covariates tested
☐	☒	A description of any assumptions or corrections, such as tests of normality and adjustment for multiple comparisons
☐	☒	A full description of the statistical parameters including central tendency (e.g. means) or other basic estimates (e.g. regression coefficient) AND variation (e.g. standard deviation) or associated estimates of uncertainty (e.g. confidence intervals)
☒	☐	For null hypothesis testing, the test statistic (e.g. *F*, *t*, *r*) with confidence intervals, effect sizes, degrees of freedom and *P* value noted<br>*Give P values as exact values whenever suitable.*
☒	☐	For Bayesian analysis, information on the choice of priors and Markov chain Monte Carlo settings
☒	☐	For hierarchical and complex designs, identification of the appropriate level for tests and full reporting of outcomes
☒	☐	Estimates of effect sizes (e.g. Cohen's *d*, Pearson's *r*), indicating how they were calculated

Our web collection on statistics for biologists contains articles on many of the points above.

Software and code

Policy information about availability of computer code

Data collection	BD FACSDiva<br>ID7000 Software 2.2.1.17271<br>NIS-Elements AR 6.10<br>Gen5 3.12 Software
Data analysis	FlowJo v10.10<br>GraphPad Prism 10<br>RStudio 4.4.2<br>Morpheus<br>LEGENDplex™ Data Analysis Software<br>Image J v1. 54f<br>QuPath 0.4.4<br>ID7000 Software 2.2.1.17271<br>Living Image 4.8.2 Software

For manuscripts utilizing custom algorithms or software that are central to the research but not yet described in published literature, software must be made available to editors and reviewers. We strongly encourage code deposition in a community repository (e.g. GitHub). See the Nature Portfolio guidelines for submitting code & software for further information.

Data

Policy information about availability of data

All manuscripts must include a data availability statement. This statement should provide the following information, where applicable:

- Accession codes, unique identifiers, or web links for publicly available datasets
- A description of any restrictions on data availability
- For clinical datasets or third party data, please ensure that the statement adheres to our policy

Data supporting the findings of this study are available within the Article, Supplementary Information or Source Data file. Source data are provided as a Source Data file.

Research involving human participants, their data, or biological material

Policy information about studies with human participants or human data. See also policy information about sex, gender (identity/presentation), and sexual orientation and race, ethnicity and racism.

Reporting on sex and gender	*Use the terms sex (biological attribute) and gender (shaped by social and cultural circumstances) carefully in order to avoid confusing both terms. Indicate if findings apply to only one sex or gender; describe whether sex and gender were considered in study design; whether sex and/or gender was determined based on self-reporting or assigned and methods used. Provide in the source data disaggregated sex and gender data, where this information has been collected, and if consent has been obtained for sharing of individual-level data; provide overall numbers in this Reporting Summary. Please state if this information has not been collected. Report sex- and gender-based analyses where performed, justify reasons for lack of sex- and gender-based analysis.*
Reporting on race, ethnicity, or other socially relevant groupings	*Please specify the socially constructed or socially relevant categorization variable(s) used in your manuscript and explain why they were used. Please note that such variables should not be used as proxies for other socially constructed/relevant variables (for example, race or ethnicity should not be used as a proxy for socioeconomic status). Provide clear definitions of the relevant terms used, how they were provided (by the participants/respondents, the researchers, or third parties), and the method(s) used to classify people into the different categories (e.g. self-report, census or administrative data, social media data, etc.) Please provide details about how you controlled for confounding variables in your analyses.*
Population characteristics	*Describe the covariate-relevant population characteristics of the human research participants (e.g. age, genotypic information, past and current diagnosis and treatment categories). If you filled out the behavioural & social sciences study design questions and have nothing to add here, write "See above."*
Recruitment	*Describe how participants were recruited. Outline any potential self-selection bias or other biases that may be present and how these are likely to impact results.*
Ethics oversight	*Identify the organization(s) that approved the study protocol.*

Note that full information on the approval of the study protocol must also be provided in the manuscript.

Field-specific reporting

Please select the one below that is the best fit for your research. If you are not sure, read the appropriate sections before making your selection.

☒ Life sciences ☐ Behavioural & social sciences ☐ Ecological, evolutionary & environmental sciences

For a reference copy of the document with all sections, see nature.com/documents/nr-reporting-summary-flat.pdf

Life sciences study design

All studies must disclose on these points even when the disclosure is negative.

Sample size	No sample-size calculation was performed. Similarly to previous works, for flow cytometric analyses, between 3 and 5 mice per group were used in each experiment, depending on animal availability. For survival experiments, 6 to 8 mice per group were used. These sample sizes were considered sufficient to ensure reproducibility and detect consistent biological trends across experiments.
Data exclusions	Data identified as outliers by GraphPad were excluded from the analysis.
Replication	Key findings of the manuscript are supported by experiments that were independently repeated between two and four times, confirming the robustness and reproducibility of the results. The precise number of biological or technical replicate for each experiment is specified within the figure legends.
Randomization	Mice were randomized the day of the injection between the groups using the Mix&pick software of the pasteur institute (https://mixnpick.pasteur.fr/).
Blinding	Investigators were not blinded to group allocation as experimental procedures and analysis were performed by the same investigators.

Reporting for specific materials, systems and methods

We require information from authors about some types of materials, experimental systems and methods used in many studies. Here, indicate whether each material, system or method listed is relevant to your study. If you are not sure if a list item applies to your research, read the appropriate section before selecting a response.

Materials & experimental systems

n/a	Involved in the study
☐	☒ Antibodies
☐	☒ Eukaryotic cell lines
☒	☐ Palaeontology and archaeology
☐	☒ Animals and other organisms
☒	☐ Clinical data
☒	☐ Dual use research of concern
☒	☐ Plants

Methods

n/a	Involved in the study
☒	☐ ChIP-seq
☐	☒ Flow cytometry
☒	☐ MRI-based neuroimaging

Antibodies

Antibodies used

Mouse antibodies
CD11b AF700 (M1/70) Biolegend Cat# 101222, RRID:AB_493705 200
CD11b BV421 (M1/70) Biolegend Cat# 101236, RRID:AB_11203704 200
CD11b FITC (M1/70) Biolegend Cat# 101206, RRID:AB_312789 200
CD11b BUV395 (M1/70) BD Cat# 565976, RRID:AB_2721166 200
CD11b BV785 (M1/70) Biolegend Cat# 101243, RRID:AB_2561373 200
CD11b StarBright Blue 675 (5C.6) Bio-Rad Cat#MCA711SBB675, RRID:AB_3101464 200
CD11c PeCy7 (N418) Biolegend Cat# 117318, RRID:AB_493568 200
CD11c PE/Dazzle™ 594 (N418) Biolegend Cat# 117348, RRID:AB_2563655 200
CD16/32 BV510 (93) Biolegend Cat# 101333, RRID:AB_2563692 200
CD24 BV510 (M1/69) BioLegend Cat# 101831, RRID: AB_2563894 400
CD26 PeCy7 (H194-112) Biolegend Cat# 137810, RRID:AB_2564312 200
CD3e PE (145-2c11) Biolegend Cat# 100308, RRID:AB_312673 100
CD4 BV510 (RM4-5) Biolegend Cat# 100559, RRID:AB_2562608 400
CD4 PercpCy5.5 (RM4-5) Biolegend Cat# 100540, RRID:AB_893326 400
CD4 AF700 (RM4-5) Biolegend Cat# 100536, RRID:AB_493701 400
CD44 APC/Fire750 (IM7) Biolegend Cat# 103062, RRID:AB_2616727 200
CD45 BUV395 (30-F11) eBioscience Cat# 363-0451-82, RRID:AB_2925264 800
CD45 BUV737 (30-F11) eBioscience Cat# 367-0451-82, RRID: AB_2895963 800
CD45 APC/Fire750 (30F11) Biolegend Cat# 103154, RRID:AB_2572116 800
CD45 RB613 (I3/2) BD Cat # 758191, RRID:AB_3690340 400
CD45 AF700 (30-F11) Biolegend Car# 103128, RRID:AB_493715 800
CD45 BUV805 (30F11) BD Cat# 568336, RRID:AB_3684191 800
CD45.1 PE-Cy7 (A20) Biolegend Cat# 110729, RRID:AB_1134170 600
CD45.1 BV605 (A20) Biolegend Cat# 110738, RRID:AB_2562565 600
CD45.2 PerCp-Cy5 (104) eBioscience Cat# 45-0454-82 RRID: AB_953590 200
CD45RB APC (C363-16A) BioLegend Cat# 103319; RRID: AB_2565228 200
CD49a AF647 (Ha31/8) BD Cat# 562113, RRID:AB_11153312 200
CD62L FITC (MEL-14) Biolegend Cat# 104406, RRID:AB_313093 200
CD62L BV785 (MEL-14) Biolegend Cat# 104440, RRID:AB_2629685 200
CD64 BV711 (X54-5/7.1) Biolegend Cat# 139311, RRID:AB_2563846 200
CD80 BV421 (16-10A1) Biolegend Cat# 104725, RRID:AB_10900989 400
CD8a (KT15) FITC BioRad Cat# MCA609G, RRID: AB_321407 400
CD8a BV785 (53-6.7) Biolegend Cat# 100750, RRID:AB_2562610 400
CD8b BUV395 (H35-17.2) BD Cat# 740278, RRID:AB_2740017 400
CX3CR1 BV785 (SA011F11) Biolegend Cat# 149029, RRID:AB_2565938 200
Ki67 APC (SolA15) Invitrogen Cat# 56-5698-82, RRID:AB_2637480 200
Ly6C APC/ Fire750 (HK1.4) Biolegend Cat# 128046, RRID:AB_2616731 200
Ly6C BUV805 (HK1.4) BD Cat# 755202, RRID:AB_3687609 200
Ly6G BV650 (1A8) Biolegend Cat# 127641, RRID:AB_2565881 400
I-A/I-E APC (M5/114.15.2) Biolegend Cat# 107614, RRID:AB_313329 400
I-A/I-E AF700 (M5/114.15.2) Biolegend Cat# 107622, RRID:AB_493727 400
PD-1 (CD279) BV711 (29F.1A12) Biolegend Cat# 135231, RRID:AB_2566158 200
SiglecH BUV395 BD Cat# 747669, RRID: AB_2744230 200
SiglecF BV421 (E50-2440) BD Cat# 562681, RRID:AB_2722581 400
TCRb BV650 (H57-597) Biolegend Cat# 109251, RRID:AB_2810348 400

Tim3 PE (RMT3-23) BD Cat# 568428; RRID: AB_345377 200
PD-L1 BV605 Biolegend Cat# 124321, RRID:AB_2563635 400
XCR1 PE (ZET) Biolegend Cat# 148204, RRID:AB_2563843 200
XCR1 BV785 (ZET) Biolegend Cat# 148225, RRID:AB_2783119 200
NK1.1 PE-Cy7 (clone S17016D) Biolegend Cat# 156513; RRID:AB_2888852 200
CD40 PE-Dazzle584 (3/23) Biolegend Cat# 124629; RRID: AB_2572184 200
CD40 RY610 (3/23) BD Cat# 759052, RRID:AB_3691150 200
CD86 FITC (GL-1) Biolegend Cat# 105005, RRID: AB_313148 200
CD86 PE (GL-1) Biolegend Cat# 105008, RRID:AB_313151 200
CD86 AF700 (GL-1) Biolegend Cat#105023, RRID:AB_493720 200
NKp46 BV421 (29A1.4) Biolegend Cat# 137612, RRID:AB_2563104 200
EpCAM BV510 (G8.8) Biolegend Cat# 101831; RRID: AB_2563894 400
Podoplanin/gp38 BV421 (8.1.1) Biolegend Cat#127423 ; RRID: AB_2814017 200
CD31 BV785 (390) Biolegend Cat#102435 ; RRID: AB_2810334 400
ICAM-1 PE (YN1/1.7.4) Biolegend Cat#116107 ; RRID: AB_313698 400
IFNγ BV785 (XMG1) Biolegend Cat# 505837; RRID: AB_2629667 100
IFNγ BV650 (XMG1) Biolegend Cat# 505831; RRID: AB_11142685 100
TCF1/TCF7 PE (S33-966) BD Cat# 564217; RRID: AB_2687845 100
Foxp3 FITC (FJK-16S) eBioscience Cat# 11-5773-82 ; RRID: AB_465243 200
CD172a PE-Cy7 (P84) Biolegend Cat# 144007; RRID: AB_2563545 200
CD172a APC (P84) BD Cat# 560106, RRID:AB_1645218 200
Streptavidin APC-R700 BD Cat# 565144, RRID:AB_2869657 800
FLT3 PE (A2F10) eBioscience Cat# 12-1351-82, RRID: AB_465859 200
ESAM PE (1G8/ESAM) Biolegend Cat# 136203; RRID: AB_1953300 200
TNFα PE-Cy7 (MP6-XT22) Biolegend Cat# 506305; RRID: AB_315426 100
IL2 PE-Cy5 (JES6-5H) Biolegend Cat# 503824; RRID: AB_2123674 100
Granzyme B FITC (QA16A02) ebioscience Cat# 11-8898-82; RRID: AB_10733414 100
CD4 PerCP-Cy5.5 (GK1.5) Biolegend Cat# 100434; RRID: AB_893324 400
CD73 PE (eBioTY/11.8) Biolegend Cat# 127205; RRID: AB_1089065 100
CD90.2 APC (30–H12) Biolegend Cat# 105311; RRID: AB_313182 100
CD140a APC (APA5) Biolegend Cat# 135908; RRID: AB_2043970 100
CCR5 APC (HM-CCR5) Biolegend Cat# 107011; RRID: AB_2074528 100
CXCR3 (S18001A) PE Biolegend Cat# 155903; RRID: AB_2783130 100
Anti-mouse CD28, (37.51), Ultra-LEAF™ Biolegend Cat# 102116; RRID: AB_11147170 2000
Anti-mouse CD3e (17A2) Biolegend Cat# 100243; RRIB: AB_2563946 200
Anti-mouse IL-4 (clone 11B11) Ultra-LEAF™ Biolegend Cat# 504122, RRID: AB_11149679 200
InVivoMAb rat IgG2b isotype control, anti-keyhole limpet hemocyanin (clone LTF-2) BioXcell Cat# BE0090; RRID: AB_1107780 See Methods section
InVivoMAb anti-mouse agonist PD-1 (B7-H1) BioXcell Cat# BE0273; RRID: AB_10949073
Lot: 883023J1 See Methods section
InVivoMAb anti-mouse agonist CTLA-4 (CD152, Clone 9H10) BioXcell Cat# BE0131; RRID: AB_10950184
Lot: 834323S1 See Methods section
InVivoMab anti-mouse CD4 (GK1.5) BioXcell Cat# BE0003-1; RRID: AB_1107636
Lot: 805422A1 See Methods section
InVivoMab anti-mouse CD8 (2.43) BioXcell Cat# BE0061; RRID: AB_1125541
Lot: 811522A2 See Methods section
InVivoMab anti-mouse NK1.1 (PK136) BioXcell Cat# BE0036; RRID: AB_1107737
Lot: 796521N1 See Methods section
InVivoMAb anti-mouse CD25 (PC-61.5.3) BioXcell Cat# BE0012; RRID:AB_1107619
Lot:795321D1 See Methods section
InVivoMAb anti-mouse CD40 (FGK4.5) BioXcell Cat# BE0016-2; RRID:AB_1107647
Lot: 805122F1 See Methods section
CD45 Biotin (30-F11) Biolegend Cat# 103103; RRID:AB_312968 400
Ly6G Biotin (1A8) Biolegend Cat# 127604; RRID: AB_1186105 400
CD3 Biotin (145-2C11) Biolegend Cat# 100304; RRID: AB_312669 400
CD19 Biotin (6D5) Biolegend Cat# 115503; RRID: AB_313638 400
CD45R/B220 Biotin (RA3-6B2) Biolegend Cat# 103203; RRID: AB_312988 400
NK1.1 Biotin (PK136) Biolegend Cat# 108703; RRID: AB_313390 400
SiglecF Biotin (F17007L) Biolegend Cat# 155512; RRID: AB_2814066 400
TER119 Biotin (TER-119) Biolegend Cat# 116203; RRID: AB_313704 400
CD11b Biotin (M1/70) Biolegend Cat# 101204, RRID:AB_312787 400
I-A/I-E Biotin (M5/114.15.2) Biolegend Cat# 107604, RRID:AB_313319 400
Human antibodies
Human FLT3L Biotin (Polyclonal) Bio-Techne/R&D systems Cat# BAF308; RRID:AB_2278494 100
NKp46 Biotin (9E2) Biolegend Cat#331906, RRID:AB_1027671 40
CD3 Biotin (OKT3) Biolegend Cat#317319, RRID:AB_10918432 400
CD19 Biotin (HIB19) Biolegend Cat#302203, RRID:AB_314233 400
CD56 Biotin (HCD56) Biolegend Cat#318319, RRID:AB_893392 40
CD66b Biotin (G10F5) Biolegend Cat#305120, RRID:AB_2566608 40

CD203c Biotin (REA826) Miltenyi Cat#130-112-811, RRID:AB_2656166 100
CD20 Biotin (2H7) Biolegend Cat#302349, RRID:AB_2565523 200
HLA-DR BUV737 (L243) BD Cat#753688, RRID:AB_3687372 100
CD163 BUV661 (GHI/61) BD Cat#741645, RRID:AB_2871044 100
BDCA-2 BUV615 (V24-785) BD Cat#751078, RRID:AB_2875114 100
CD16 BUV395 (3G8) BD Cat#563785 100
BTLA BV421 (MIH26) Biolegend Cat#344511, RRID:AB_2566507
CD5 BV605 (L17F12) Biolegend Cat#364019, RRID:AB_2565940 100
CD123 BV650 (6H6) Biolegend Cat#306019, RRID:AB_11218792 100
CD141 BV711 (M80) Biolegend Cat#344135, RRID:AB_3097423 40
CD14 BV786 (M5E2) BD Cat#563699 100
AXL FITC (108724) RnD Systems Cat#18624513 100
XCR1 PerCP-Cy5.5 (S15046E) Biolegend Cat#372629, RRID:AB_2924564 100
CLEC10A PE (H037G3) Biolegend Cat#354703, RRID:AB_11219202 100
CD88 PE-DAZZLE (S5/1) Biolegend Cat#344317, RRID:AB_2750446 100
CD45 PE-Cy5 (HI30) BD Cat#555484, RRID:AB_395876 200
CD1c PE-Cy7 (L161) Biolegend Cat#331515, RRID:AB_1953227 100
CLEC9A APC (8F9) Biolegend Cat#353805, RRID:AB_2565518 100
CX3CR1 R718 (2A9-1) BD Cat#752200, RRID:AB_2917307 100
CD45RA APC-Cy7 (HI100) Biolegend Cat#304127, RRID:AB_10708419 100

Validation

All antibodies were used according to manufacturer's instructions: Biolegend (www.biolegend.com), BD (https://www.bdbiosciences.com/en-fr), eBioscience (www.thermofisher.com), BioXCell (https://bioxcell.com), R&D Systems (www.rndsystems.com), Miltenyi Biotec (https://www.miltenyibiotec.com/FR-en/), Bio-Rad (https://www.bio-rad-antibodies.com)

Working dilutions were previously established in the lab or identified using positive controls (cells supposed to express the target).

Eukaryotic cell lines

Policy information about cell lines and Sex and Gender in Research

Cell line source(s)

Only murine cell lines were used in this paper. B16F10 (male, DOI: 10.1016/j.immuni.2020.06.002), B16-huFLT3L-GFP (OI: 10.1016/j.immuni.2020.06.002), B16-OVA, YUMM-OVA (male), E0771 (female, Dr. Stéphanie Hugues, ATCC Number: CRL-3461), TC-1 (Dr. Alexandre Boissonnas), MC38 (female, Dr. Philippe Bousso,Cat# SCC172 (Sigma-Aldrich)), mesenchymal stromal cells (female, Dr. Loredana Saveanu).

Authentication

None of the cell lines were authenticated.

Mycoplasma contamination

All cell lines were tested negative for mycosplasma detection.

Commonly misidentified lines
(See ICLAC register)

None.

Animals and other research organisms

Policy information about studies involving animals; ARRIVE guidelines recommended for reporting animal research, and Sex and Gender in Research

Laboratory animals

mouse: C57BL/6J Janvier Labs RRID:IMSR_JAX:000664
mouse: CD45.1 (CByJ.SJL(B6) Ptprca/J) Janvier Labs RRID:IMSR_JAX:006584
mouse: OT-1 Rag2-/- CD45.1 Dr. Sebastian Amigorena DOI: 10.1038/s41467-022-31504-z
mouse: Rosa-DTA (B6.129P2 Gt(ROSA)26Sortm1(DTA)Lky/J) Dr. Marc Dalod RRID:IMSR_JAX:009669
mouse: ROSA26-LSL-RFP (B6.Cg Gt(ROSA)26Sortm1Hjf/J) Dr. Marc Dalod RRID:IMSR_JAX:038164
mouse: Xcr1-Cre (B6 Xcr1tm1Ciphe) Dr. Marc Dalod DOI: 10.3389/fimmu.2018.02805
mouse: ROSA26-LSL-tdTomato (B6.Cg Gt(ROSA)26Sortm14(CAG-tdTomato)Hze/J) Dr. Tessa Bergsbaken RRID:IMSR JAX:007914
mouse: Irf8-GFP (B6.Cg Irf8tm2.1Hm/J) The Jackson Laboratory RRID: IMSR_JAX:027084
Both males and females were used between 8 and 12 weeks of age.

mouse: BALB/c Rag2-/- IL2rγ-/- SirpαNOD (BRGS), BALB/c Rag2-/- IL2rγ-/- SirpαNOD Flt3+/+ Human Disease Model Core facility - Pasteur Institute DOI : 10.1002/eji.201848001
mouse: BALB/c Rag2-/- IL2rγ-/- SirpαNOD (BRGS), BALB/c Rag2-/- IL2rγ-/- SirpαNOD Flt3-/- Human Disease Model Core facility - Pasteur Institute DOI : 10.1002/eji.201848001
Both males and females were at 16 weeks of age.

Wild animals

The study did not involve wild animals.

Reporting on sex

We found no sex-related differences. Therefore, both male and female mice were used depending on the experiment and the tumor model, as described in the Methods section.

Field-collected samples	The study did not involve samples collected from the field.
Ethics oversight	The study was approved by the local ethics committee (Comité d'éthique Paris Nord n°121 or CEEA89) and the French Ministry of Education and Research under the authorization number APAFIS#15373 and APAFIS #48865. Animal care and treatment were conducted with national and international laws and policies (European Economic Community Council Directive 86/609; OJL 358; December 12, 1987). All experiments were performed in accordance with the Federation of European Laboratory Animal Science Association (FELASA) guidelines, institutional guidelines and the French law.

Note that full information on the approval of the study protocol must also be provided in the manuscript.

Plants

Seed stocks	*Report on the source of all seed stocks or other plant material used. If applicable, state the seed stock centre and catalogue number. If plant specimens were collected from the field, describe the collection location, date and sampling procedures.*
Novel plant genotypes	*Describe the methods by which all novel plant genotypes were produced. This includes those generated by transgenic approaches, gene editing, chemical/radiation-based mutagenesis and hybridization. For transgenic lines, describe the transformation method, the number of independent lines analyzed and the generation upon which experiments were performed. For gene-edited lines, describe the editor used, the endogenous sequence targeted for editing, the targeting guide RNA sequence (if applicable) and how the editor was applied.*
Authentication	*Describe any authentication procedures for each seed stock used or novel genotype generated. Describe any experiments used to assess the effect of a mutation and, where applicable, how potential secondary effects (e.g. second site T-DNA insertions, mosiacism, off-target gene editing) were examined.*

Flow Cytometry

Plots

Confirm that:

- [x] The axis labels state the marker and fluorochrome used (e.g. CD4-FITC).
- [x] The axis scales are clearly visible. Include numbers along axes only for bottom left plot of group (a 'group' is an analysis of identical markers).
- [x] All plots are contour plots with outliers or pseudocolor plots.
- [x] A numerical value for number of cells or percentage (with statistics) is provided.

Methodology

Sample preparation	Murine tumors, tumor-draining lymph nodes (LNs), spleens, or synthetic niches were harvested and subjected to enzymatic digestion. Tissues were incubated in 3mL of digestion buffer, comprising Hank's Balanced Salt Solution (HBSS) supplemented with calcium and magnesium (Thermo Fisher), 75µg/mL Collagenase D (Roche), and 0.02mg/mL DNase I (Thermo Fisher), at 37°C for 30 minutes. Single-cell suspensions were then generated by smashing the digested tissues through 70µm cell strainers (Corning). Spleen red blood cells were lysed using ACK lysis buffer (ThermoFisher).
Instrument	Data acquisition were performed using a BD LSR-Fortessa X20, a BD Symphony A5 (BD Biosciences), or a SONY ID7000.
Software	Analysis were done using FlowJo software or the ID7000 software.
Cell population abundance	Purity of FACS-sorted samples was assessed at the sorter and frequency of sorted cells was above 95% of live cells.
Gating strategy	Gating strategies are specified throughout the figures or supplementary materials.

- [x] Tick this box to confirm that a figure exemplifying the gating strategy is provided in the Supplementary Information.
